# ERG is specifically associated with ETS-2 and ETV-4, but not with ETS-1, in prostate cancer

**DOI:** 10.3892/ijmm.2012.1097

**Published:** 2012-08-20

**Authors:** DAVID ADLER, JACQUELINE OCHSENFAHRT, KERSTIN FUCHS, GLEN KRISTIANSEN, SVEN PERNER, NICOLAS WERNERT

**Affiliations:** 1 Institute of Pathology and; 2 Department of Prostate Cancer Research, Institute of Pathology, University Hospital of Bonn, D-53127 Bonn, Germany

**Keywords:** ETS-related gene, ETS-2, ETS variant gene-4, ETS-1, prostate cancer

## Abstract

The erythroblast transformation-specific (ETS) family of transcription factors plays important roles in both physiological and pathological conditions. Even though many studies have focused on single ETS factors within a single tissue and within the context of specific promoters, the functional impact of multiple ETS members present within a specific cell type has not yet been investigated, especially in prostate cancer (PCa). As the most prominent gene rearrangement in PCa leads to the overexpression of the ETS-related gene (ERG), the aim of this study was to investigate whether ERG is part of a complex integrated transcriptional network that involves other ETS factors. More specifically, as the ETS family consists of 27 members, we focused our efforts initially on investigating whether ERG is associated with the three family members, ETS-1, ETS-2 and ETS variant gene-4 (ETV-4), in PCa as a proof of principle. Using western blot analysis, we show that ERG, ETS-1, ETS-2 and ETV-4 are expressed in PC3 cell nuclear extracts and in protein lysates prepared from human PCa prostatectomy specimens. Immunoprecipitations using an anti-ERG antibody were used with PC3 cell nuclear extracts as well as with a pooled protein lysate sample prepared from the PCa tissue samples of five patients. Importantly, our results revealed that ERG is specifically associated with ETS-2 and ETV-4, but not with ETS-1, in PC3 cell nuclear extracts and PCa tissue protein lysates. Our findings strongly support the notion that ERG is part of a complex integrated transcriptional network that involves other ETS factors, which are likely to cooperate or influence the activity of ERG in PCa. The functional impact of multiple ETS factors being associated with ERG in PCa requires further study, as it may provide insights into the mechanism by which ERG exerts its influence in PCa and may subsequently contribute to our understanding of the molecular basis of PCa.

## Introduction

Prostate cancer (PCa) is a leading cause of cancer mortality and is among the most common tyeps of cancer in Western countries (1). In PCa, recurrent gene fusions occurring between the transmembrane protease, serine 2 (*TMPRSS2)* gene, an androgen-regulated prostate-specific serine protease and members of the erythroblast transformation-specific (ETS) family of transcription factors [ETS variant gene (*ETV)-1*, *ETV-4*, *ETV-5* and most commonly, ETS-related gene *ERG*] resulting in the increased expression of the latter rearranged ETS members in response to androgens, are frequently found (2–4).

The ETS family of transcription factors which is comprised of 27 members in humans (5) has been reported to be involved in many processes such as development, differentiation, proliferation, apoptosis, migra tion, tissue remodeling, invasion and angiogenesis in a wide range of cells (such as B-cells, endothelial cells, fibroblasts, as well as neoplastic cells) (6–13). The ETS family is defined by the presence of an evolutionary conserved DNA-binding domain, termed the ETS domain, which is comprised of approximately 80 amino acids with four tryptophan repeats and recognizes DNA sequences with a GGAA/T core motif (14,15). Phylogenetic analysis of the human ETS domains has led to the identification of subfamilies of more highly related members (16).

Even though different ETS family members may have different functions due to their binding preferences for distinct flanking sequences around the GGAA/T core motif enabling them to bind more specifically (16), overlapping func tions of ETS members and redundant occupancy at gene regions have been described (16,17). Furthermore, even though the ETS transcription factors may have independent activities, it is likely that they are part of an integrated network, in which gene regulation may be influenced by the binding equilibrium and the activity of the ETS transactivation domains, as well as the complex formation of different ETS members and other factors (18,19).

While a number of studies have focused on single ETS factors within a single tissue and within the context of specific promoters, the functional impact of multiple ETS members present within a specific cell type have not yet been investigated (18), particularly in PCa. While multiple ETS factors may be able to regulate the same sets of genes, the magnitude or the directions may be different for each of the ETS factors (18).

As the most prominent gene rearrangement in PCa leads to the overexpression of ERG (20–23), combined with functional studies showing that *ERG* knockdown induces morphological changes, inhibits cell growth in both culture and mice, and that *ERG* overexpression leads to an increase in cell invasion (24), the aim of the present study was to investigate whether ERG is part of a complex integrated transcriptional network that involves other ETS factors which are highly likely to cooperate or influence the activity of ERG in PCa.

More specifically, as the ETS family of transcription factors consists of 27 members (5), we decided to focus our efforts initially on investigating whether ERG is associated with three well-known members of the family, ETS-1, ETS-2 and ETV-4, in PCa as a proof of principle. The rationale behind choosing the latter ETS members was that ETS-1, the prototype of the ETS family, is overexpressed in latent as well as clinically manifest PCa (25), ETS-2 is also overexpressed in PCa (18), ETS-2 and ETS-1 play redundant roles (17), ETS-2 is associated with *in vitro* synthesized ETS-1 (26), ETS-2 interacts with ERG *in vivo* demonstrated by the two-hybrid system (26) and that ETV-4 is rearranged in PCa, similar to ERG (2–4). The results from a previous study were also taken into account, namely that the occurrence of multiple ETS rearrangements within one prostate gland, within the same tumor focus and within the same nucleus (27).

## Materials and methods

### Western blot analysis

The expression of ERG, ETS-1, ETS-2 and ETV-4 in PC3 cell nuclear extracts (Santa Cruz Biotechnology, Inc., Santa Cruz, CA, USA) was determined by western blot analysis using a mouse monoclonal anti-ERG antibody (Bio-Care, Holt, MI, USA), a mouse monoclonal anti-ETS-1 antibody (Transduction Laboratories, Lexington, KY, USA), a rabbit polyclonal anti-ETS-2 antibody (Sigma-Aldrich, Munich, Germany) and a mouse monoclonal anti-ETV-4 antibody (BioCat, Heidelberg, Germany), respectively.

In protein lysates prepared from human PCa prostatectomy specimens of five patients, the expression of ERG, ETS-1, ETS-2 and ETV-4 was determined by western blot analysis using a mouse monoclonal anti-ERG antibody (Biocare), a mouse monoclonal anti-ETS-1 antibody (Transduction Laboratories) and a rabbit polyclonal C-20 anti-ETS-1 antibody (Santa Cruz Biotechnology, Inc.), a rabbit polyclonal anti-ETS-2 antibody (Sigma-Aldrich) and a mouse monoclonal anti-ETV-4 antibody (BioCat), respectively.

### Human PCa prostatectomy specimens and protein lysate preparations

Briefly, fresh tissue samples from five patients with prostate carcinomas (Gleason scores, 6, 6 7, 7 and 8) were taken immediately after radical prostatectomy. The tissue samples were then shock-frozen in liquid nitrogen with ice-cold isopentane as described previously (28). Thereafter, 6-μm-thick frozen sections were cut from the samples using a cryotome (Leica, Berlin, Germany) and mounted on conventional slides followed by staining with hematoxylin and eosin (H&E) for diagnostic evaluation by an experienced pathologist. The preparation of protein lysates from the latter prostate carcinoma tissue samples was carried out as previously described (29).

### Immunoprecipitation (IP)

To investigate whether ERG is associated with ETS-1, ETS-2 and ETV-4 in PC3 cell nuclear extracts (Santa Cruz Biotechnology, Inc.), we performed IP using a rabbit polyclonal anti-ERG antibody (Gentex, Zeeland, MI, USA) as it exhibited the best compatibility with our IP compared to all the other commercially available antibodies that we tested. Briefly, PC3 cell nuclear extracts were pre-cleared with protein A agarose beads (Sigma-Aldrich) by rotation at 4°C for 2 h. An additional tube containing protein A agarose beads (Sigma-Aldrich) and the rabbit polyclonal anti-ERG antibody (Gentex) was incubated by rotation at 4°C for 2 h. Both tubes were then centrifuged at 2,000 x g for 2 min and the pre-cleared supernatant was then added to the ERG-bound protein A agarose beads. The sample was then incubated by rotation for 1 h at 4°C, collected by centrifugation at 2,000 x g for 2 min, and then washed three times with a nuclear extract buffer (20 mM Hepes, 25% glycerol, 2 M KCL, 1 mM MgCl_2_, 1% Nonidet-p40, 0.5 mM EDTA). The beads were then re-suspended in a loading buffer, boiled and loaded on an SDS/PAGE gel, followed by western blot analysis using a mouse monoclonal anti-ERG antibody (Biocare), a mouse monoclonal anti-ETS-1 antibody (Transduction Laboratories), a rabbit polyclonal anti-ETS-2 antibody (Sigma-Aldrich) and a mouse monoclonal anti-ETV-4 antibody (BioCat), respectively.

In order to investigate whether ERG is associated with ETS-1, ETS-2 and ETV-4 in the protein lysates prepared from the above five PCa prostatectomy specimens, we first pooled all the five protein lysates and then performed IP as described above. Western blot analysis was performed with the same antibodies used for the above IP and additionally with a rabbit polyclonal C-20 anti-ETS-1 antibody (Santa Cruz Biotechnology, Inc.).

## Results

### Protein expression of ERG, ETS-1, ETS-2 and ETV-4 in PC3 cell nuclear extracts and in protein lysates prepared from human PCa prostatectomy specimens

**Figure 1.**
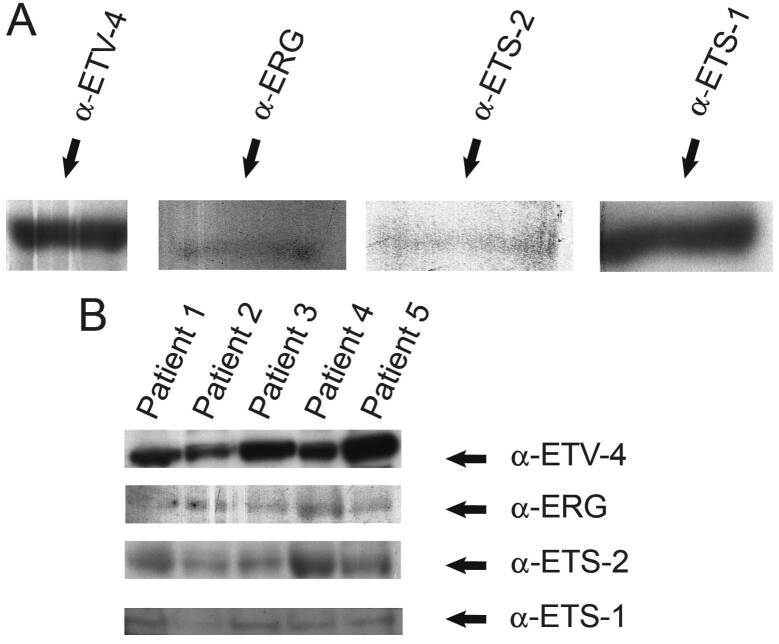
Expression of ERG, ETS-1, ETS-2 and ETV-4 in PC3 cell nuclear extracts and PCa tissues. Western blot analysis showed that ERG, ETS-1, ETS-2 and ETV-4 were expressed in (A) PC3 cell nuclear extracts, as well as in protein lysates prepared from (B) the PCa tissue samples of five patients.

As shown by western blot analysis using specific antibodies, ERG, ETS-1, ETS-2 and ETV-4 were expressed in PC3 cell nuclear extracts (Santa Cruz Biotechnology, Inc.), as well as in protein lysates prepared from the human PCa prostatectomy specimens of five patients (Fig. 1).

### ERG is specifically associated with ETS-2 and ETV-4, but not with ETS-1, in PC3 cell nuclear extracts

**Figure 2.**
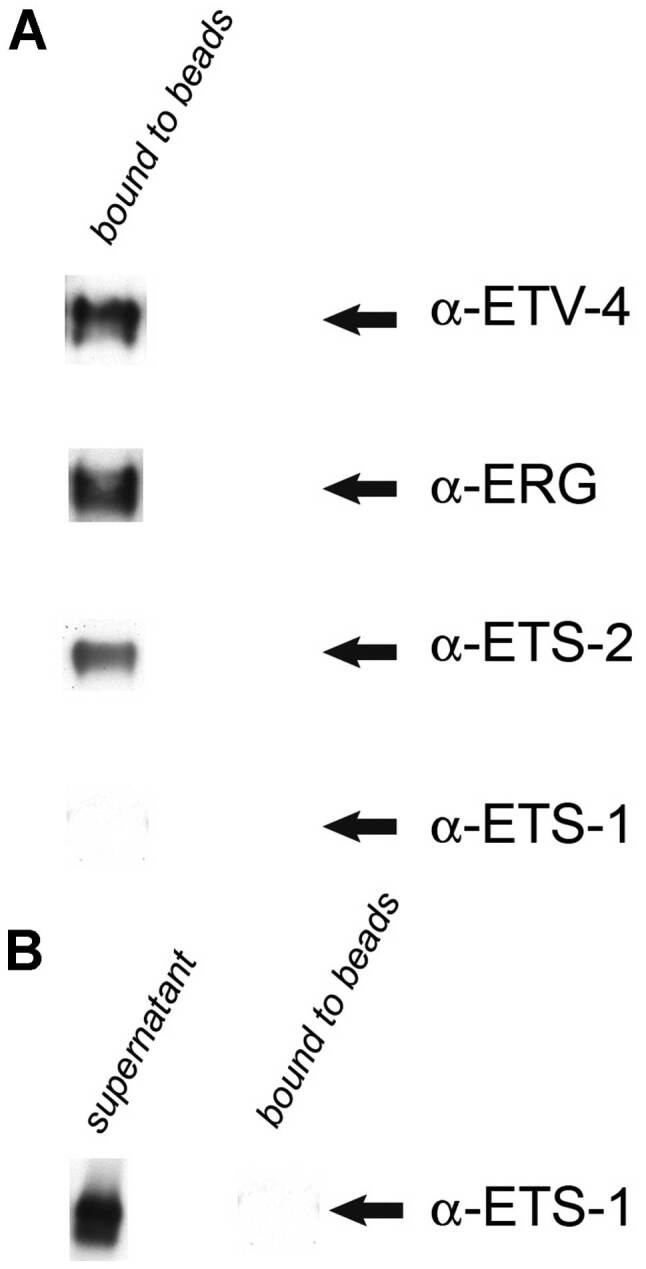
ERG is specifically associated with ETS-2 and ETV-4, but not with ETS-1, in PC3 cell nuclear extracts. Immunoprecipitation (IP) using an anti-ERG antibody showed that ERG is specifically associated with (A) ETS-2 and ETV-4, but not with (B) ETS-1, in PC3 cell nuclear extracts.

To investigate whether ERG is associated with ETS-1, ETS-2 and ETV-4 in PC3 cell nuclear extracts (Santa Cruz Biotechnology, Inc.), IP was performed using a rabbit polyclonal anti-ERG antibody (Gentex). Following western blot analysis, the results revealed that ERG is specifically associated with ETS-2 and ETV-4, but not with ETS-1 (Fig. 2).

### ERG is specifically associated with ETS-2 and ETV-4, but not with ETS-1, in protein lysates prepared from human PCa prostatectomy specimens

**Figure 3.**
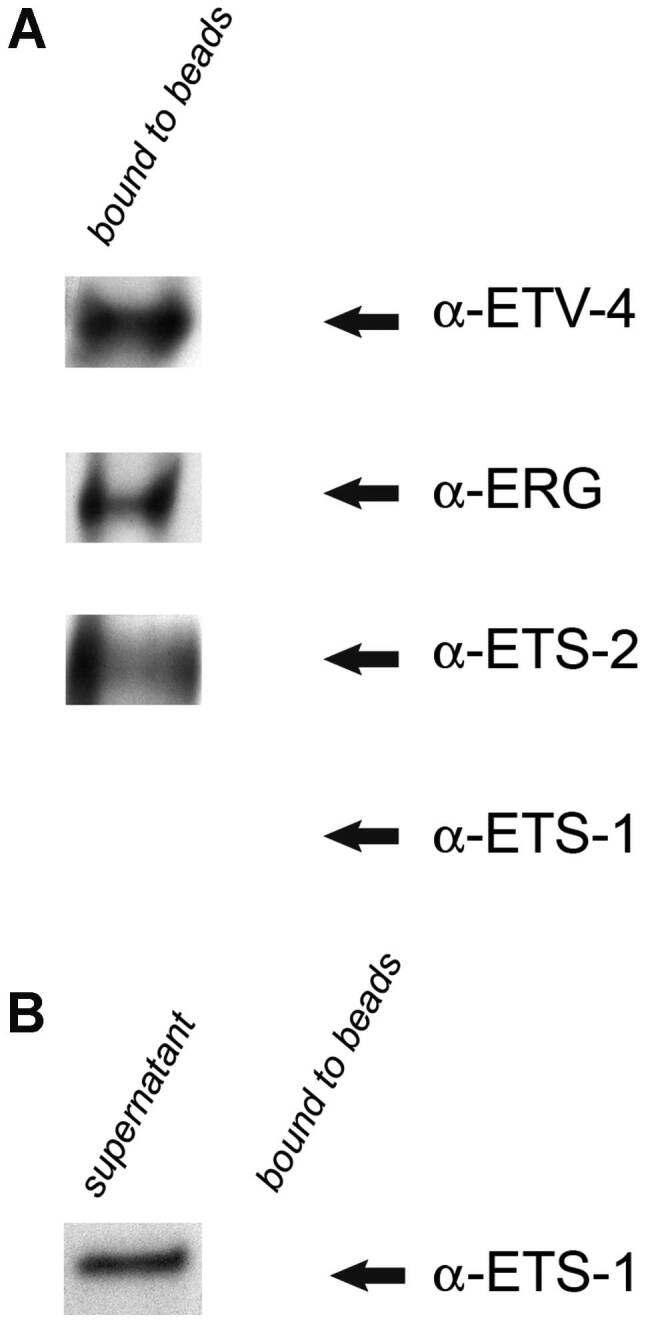
ERG is specifically associated with ETS-2 and ETV-4, but not with ETS-1, in PCa tissues. Immunoprecipitation (IP) using an anti-ERG antibody showed that ERG is specifically associated with (A) ETS-2 and ETV-4, but not with (B) ETS-1, in a pooled protein lysate sample prepared from the PCa tissue samples of five patients.

Using IP with a rabbit polyclonal anti-ERG antibody (Gentex), we investigated whether ERG is associated with ETS-1, ETS-2 and ETV-4 in a pooled protein lysate sample that was prepared from the PCa prostatectomy specimens of five patients. Following western blot analysis, the results revealed that ERG is specifically associated with ETS-2 and ETV-4, but not with ETS-1 (Fig. 3).

## Discussion

The most prominent gene rearrangement in PCa arises between the androgen-regulated prostate-specific serine protease *TMPRSS2* gene and the ETS transcription factor *ERG* gene, leading to the overexpression of ERG (20–23). The *ERG* rearrangement is highly specific to PCa (30), is maintained in advanced disease (2,31), and depending on the cohort design and the histological subtypes of PCa (20,32–34) approximately 15–80% of PCa patients harbour the *TMPRSS2-ERG* fusions (2–4,35,36). Furthermore, the knockdown of *ERG* has been reported to induce morphological changes and the inhibition of cell growth in both cell cultures and mice, whereas *ERG* overexpression has been shown to result in an increase in cell invasion (24).

Even though ETS transcription factors, such as ERG may have independent activities in PCa, ERG is highly likely to be part of a complex integrated transcriptional network that involves other ETS factors. In such a network, gene regulation may be influenced by the binding equilibrium, the activity of ETS transactivation domains and a complex formation of different ETS members and other factors, and even if multiple ETS factors may be able to regulate the same sets of genes, the magnitude or the directions may be different for each of the ETS factors (18,19).

While the focus of many studies has been on single ETS factors within a single tissue and within the context of specific promoters, the functional impact of multiple ETS factors present within a specific cell type has not yet been well investigated (18), particularly in PCa. Therefore, the aim of this study was to investigate whether ERG is associated with specific ETS transcription factors in PCa. More specifically, we investigated whether ERG is associated with ETS-1, ETS-2 and ETV-4 in PC3 cell nuclear extracts, as well as in PCa tissues.

As the ETS family of transcription factors consists of 27 members in humans (5), we decided to focus our efforts initially on investigating whether is ERG associated with three well-known members of the family, ETS-1, ETS-2 and ETV-4, in PCa as a proof of principle. The rationale behind choosing the latter ETS members was as follows: ETS-1 is the prototype of the ETS family and has been reported to be overexpressed in latent as well as clinically manifest PCa and a strong expression of ETS-1 has been associated with poor tumor differentiation (25). As shown in a previous study of our, the blockade of ETS-1 in PCa cell lines results in a decrease in cell migration (11) and has a major effect upon genes involved in the metastatic cascade (12). As ETS-1 and ETS-2 have been reported to play redundant roles during embryonic development (17), combined with reports that ETS-2 is associated with *in vitro* synthesized ETS-1 (26) and that ETS-2 interacts with ERG *in vivo* demonstrated by the two-hybrid system (26), we decided to investigate ETS-2 as well. ETS-2 has been reported to be overexpressed in PCa (18), and a blockade of ETS-2 has been shown to reduce the transformed properties of PCa cells (37), as well as growth inhibition and apoptosis (38). ETV-4 on the other hand, was chosen as *ETV-4* is known to be rearranged in PCa, similar to *ERG* (2–4), combined with a report showing the occurrence of multiple ETS rearrangements within one prostate gland, within the same tumor focus and within the same nucleus (27). Moreover, we recently reported the *ETV-4* rearranged gene status in primary PCas and their corresponding lymph nodes, and found the rearrangement in 6% of both primary PCas and the corresponding lymph node metastases (39). Furthermore, ETV-4 has been shown to be required for anchorage-independent growth and cell proliferation gene expression program in PCa cell lines (40).

Prior to investigating whether is ERG associated with ETS-1, ETS-2 and ETV-4, we first examined whether these latter proteins are expressed in PC3 cell nuclear extracts and in protein lysates prepared from human PCa prostatectomy specimens. We found by western blot analysis that ERG, ETS-1, ETS-2 and ETV-4 are expressed in both PC3 cell nuclear extracts as well as in protein lysates prepared from the PCa tissue samples of five patients (Fig. 1).

Upon confirming the expression of ERG, ETS-1, ETS-2 and ETV-4 (Fig. 1), we performed IP using an anti-ERG antibody to investigate whether ERG is associated with ETS-1, ETS-2 and ETV-4 in PC3 cell nuclear extracts, as well as in a pooled protein lysate sample prepared from the PCa tissue samples of five patients.

Of note, our results revealed that ERG is specifically associated with ETS-2 and ETV-4, but not with ETS-1, in PC3 cell, nuclear extracts (Fig. 2) and even more remarkably, we found ERG to exhibit the same specificity for ETS-2 and ETV-4, but not for ETS-1 in the pooled protein lysate sample prepared from the PCa tissue samples of five patients (Fig. 3).

Our observation that ERG is not associated with ETS-1 and ETS-2, but rather only with ETS-2, may be due to the fact that ETS-1 and ETS-2 have been reported to play redundant roles (17), which would make it counterproductive for ERG to be associated with ETS-1 and ETS-2 simultaneously. The association of ERG with ETS-2 based on our findings is supported by a previous study reporting that ERG interacts with ETS-2 *in vivo* using the two-hybrid system (26). Our finding that ERG is associated with ETV-4 is highly intriguing, as *ETV-4* is known to be rearranged in PCa, similar to *ERG* (2–4,39), combined with a recent report showing the occurrence of multiple ETS rearrangements within one prostate gland, within the same tumor focus and within the same nucleus (27), which may imply a potential cooperation or interaction between rearranged ETS genes in PCa.

Taken together, our findings that ERG is specifically associated with ETS-2 and ETV-4, but not with ETS-1, in PC3 cell nuclear extracts and PCa tissues strongly support the notion that ERG is part of a complex integrated transcriptional network that involves other ETS factors, such as ETS-2 and ETV-4, which are likely to cooperate or influence the activity of ERG in PCa. The functional impact of multiple ETS factors associating with ERG in PCa should be investigated in further studies as it may provide insights into the mechanism in which ERG exerts its influence in PCa, and subsequently contribute to our understanding of the molecular basis of PCa.
